# Relationship between obesity-related indicators and adult depression from NHANES 2021 to 2023: A cross-sectional study

**DOI:** 10.1097/MD.0000000000049216

**Published:** 2026-06-12

**Authors:** Wenting Deng, Jian Mei

**Affiliations:** a Department of Geriatrics, Jinyun County People’s Hospital, Jinyun, Zhejiang, China; b Department of Endocrinology, Jinyun County People’s Hospital, Jinyun, Zhejiang, China.

**Keywords:** BMI, depression, NHANES, obesity-related indicators, WC, WHtR, WWI

## Abstract

Obesity has been demonstrated to influence the onset and progression of depression. However, previous research has predominantly examined the association between depression and single indicators such as body mass index (BMI) or weight-adjusted waist index (WWI). While an increase in WWI and BMI correlates positively with depression scores, no studies have investigated the relationship between comprehensive obesity-related indicators – Waist-to-hip ratio (WHtR), WWI, BMI, and waist circumference (WC) – and depression scores. Using data from the National Health and Nutrition Examination Survey as the research subject, this analytical cross-sectional study used receiver operating characteristic curves, smooth curve fitting, multivariate regression analysis, and subgroup analysis to examine the association between depression, BMI, WC, WHtR, and WWI. This study included 5042 participants. WWI, WC, WHtR, and BMI showed positive correlations with depression scores (*P* < .001). There was no statistically significant difference in the relationship between BMI and depression scores according to gender and age. In the smooth curve fitting analysis, WC, WHtR, and BMI exhibited nonlinear positive correlations at inflection points of 97.6, 0.58, and 27.6, respectively. In predicting depression, the receiver operating characteristic values for WHtR, BMI, WWI, and WC showed little difference. The findings of this study indicate that body weight indicators – WWI, WC, WHtR, and BMI – are associated with depression risk. Key thresholds were identified, including WC 97.6 cm, WHtR 0.58, and BMI 27.6 kg/m^2^, and the impact of BMI on depression remained stable across different gender and age subgroups. Lifestyle interventions targeting these indicators may effectively prevent and mitigate the progression of depression.

## 1. Introduction

Depression is the leading cause of physical and mental impairment among people of all ages worldwide. Currently, at least 264 million people globally suffer from depression. Those with severe depression may lose the ability to function normally and may even choose suicide. Depression imposes a massive burden on families and causes significant socioeconomic losses. Any manifestation of depression warrants attention.^[1,2]^ Depression interacts with numerous clinical conditions, such as obesity and metabolic syndrome.^[3]^

Weight-adjusted waist index (WWI), waist circumference (WC), waist-to-height ratio (WHtR), and body mass index (BMI) are body measurements reflecting health indicators. WWI demonstrates the balance of weight and waist circumference across different BMI groups – specifically, whether waist circumference is excessive for a given weight or weight is excessive for a given waist circumference. It is considered associated with the onset of depression.^[4–6]^ WHtR correlates waist circumference with height to reflect the relative accumulation of abdominal fat relative to waist size, providing a standardized assessment of abdominal fat that addresses the inability to compare waist measurements across individuals of different heights.^[7]^ WC directly correlates with central obesity in assessing metabolic syndrome. BMI serves as a clinical indicator for evaluating human body composition, categorizing individuals as underweight, normal, overweight, or obese, offering broad applicability but failing to reflect fat distribution accurately.

The global prevalence of obesity continues to rise, affecting an increasing number of adults as a common health issue. Central obesity poses greater risks for metabolic disorders and cardiovascular diseases. Previous studies have linked obesity to depression, with most research indicating a positive correlation.^[8–11]^ This study utilized the National Health and Nutrition Examination Survey (NHANES) database from August 2021 to August 2023 to examine the relationship between depression and obesity-related indicators (WWI, WHtR, WC, and BMI).

## 2. Methods

### 2.1. Study population

**Figure 1.**
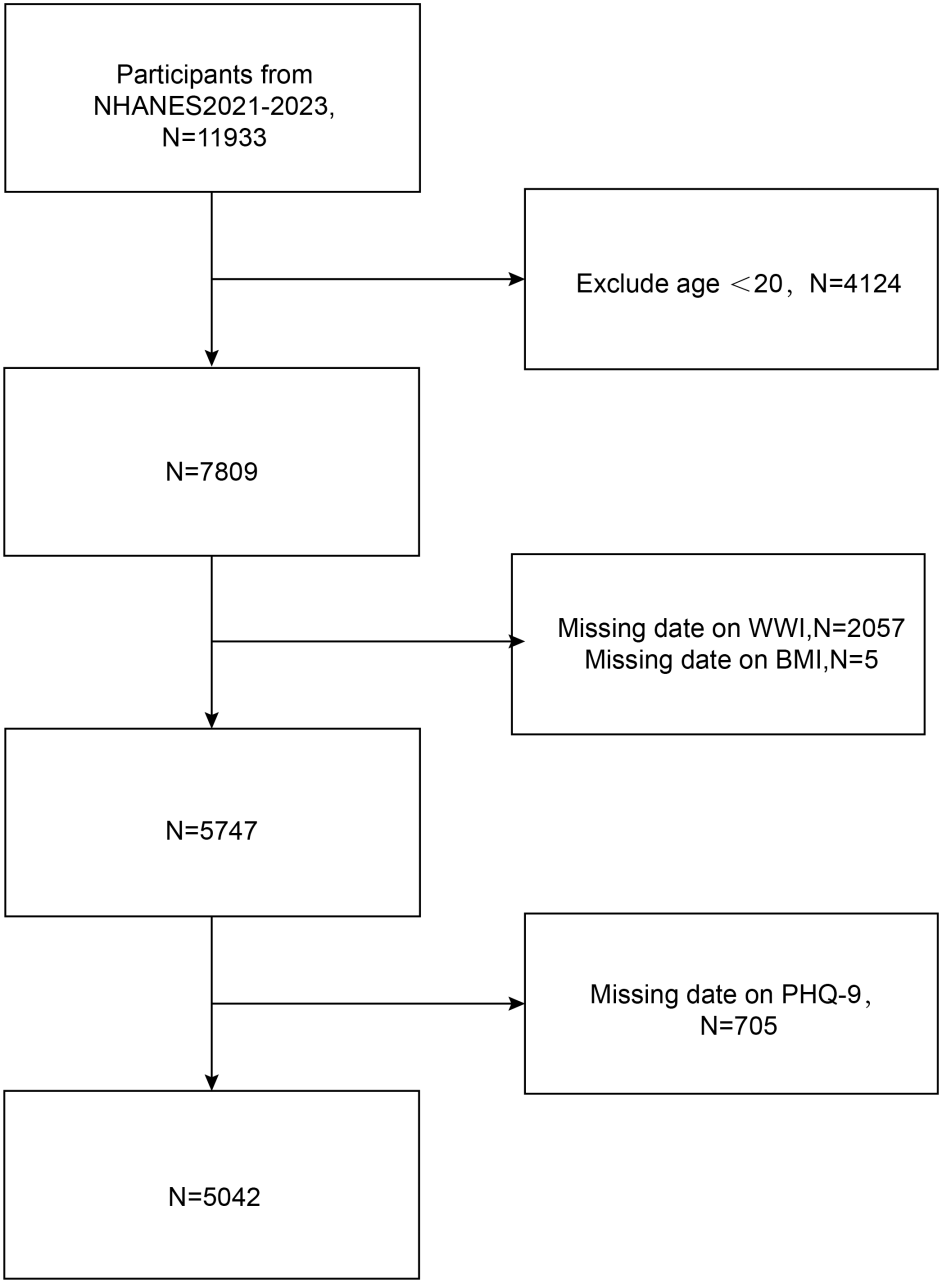
Participant selection process. BMI = body mass index, NHANES = National Health and Nutrition Examination Survey, PHQ-9 = Patient Health Questionnaire 9, WWI = weight-adjusted waist index.

This study utilized the publicly available NHANES database, a national survey conducted by the National Center for Health Statistics to evaluate the nutritional and health status of the United States, employing the dataset from August 2021 to August 2023. This paper primarily examines the relationship between adult weight-related indicators (WHtR, BMI, WWI, and WC) and depression scores. After applying inclusion and exclusion criteria, 5042 subjects were ultimately selected. Inclusion criteria: participants in the NHANES 2021 to 2023 survey; individuals aged 20 years and older; subjects must be able to understand and independently complete questionnaires. The factors for exclusion were: missing Patient Health Questionnaire 9 (PHQ-9) score data; missing weight-related indicator data (WHtR, BMI, WWI, and WC) (Fig. 1).

### 2.2. Ethics approval

In accordance with the requirements of the National Center for Health Statistics Ethics Review Committee, all participants in the NHANES study provided written informed consent.Therefore, this study does not require ethical approval.

### 2.3. Weight-related indicators

WC is measured directly, while WHtR is computed from the division of WC by height. Waist circumference is measured in centimeters, and height is measured in centimeters. Weight is divided by height squared to determine BMI. Height is expressed in meters, and weight in kilograms. Waist circumference is divided by the square root of weight to determine WWI.

### 2.4. Depression assessment

PHQ-9 consists of 9 questions. Each question is scored on a scale of 0 to 3, reflecting the frequency of symptoms over the past 2 weeks, covering 3 aspects: mood (such as feeling down and loss of interest), physical (such as poor sleep, excessive or insufficient appetite, and low energy), and cognitive (such as poor self-perception and attention disorders). Participants’ depression questionnaire data (PHQ-9) scores were used to assess depressive status: 0 to 4 points indicated no depression, 5 to 9 points indicated mild depression, 10 to 14 points indicated moderate depression, and ≥15 points indicated severe depression. Depression was defined as having a PHQ-9 score of at least 10.^[6,12]^

### 2.5. Assessment of covariates

This study included potential confounding variables affecting weight-related indicators and depression scores, encompassing categorical variables such as gender, ethnicity, marital status, education level, smoking history, drinking history, history of diabetes, and history of hypertension, as well as continuous variables including high-density lipoprotein (HDL) cholesterol, total cholesterol, fasting blood glucose, and the poverty income ratio. Consultation with a statistician determined that missing values for categorical variables were defined as negative. The statistician advised that unreported medical history could generally be reasonably inferred as “no history,” as the low proportion of missing values (<5%) would not affect results and represented a conservative approach. For education level, 1 respondent (0.02%) answered “don’t know”; for marital status, 1 respondent (0.02%) refused to answer; 3 individuals (0.06%) answered “don’t know.” For “Smoked at least 100 cigarettes in life,” 3 individuals (0.06%) refused to answer, and 4 individuals (0.08%) answered “don’t know.” For the question regarding “In the past 12 months, how often do you drink alcoholic beverages,” “Never in the last year” was defined as “no.” 2 people (0.04%) refused to answer, and 2 people (0.04%) answered “don’t know.” When asked if a doctor told them they had diabetes, 167 people (3.31%) answered “borderline.” Ever been told you had high blood pressure: 2 people (0.04%) answered “Don’t know.” You may find the measuring procedure for these variables at www.cdc.gov/nchs/nhanes.

### 2.6. Statistical analysis

Using data from the National Health and Nutrition Examination Survey (NHANES) as the research subject, continuous variables were summarized as mean ± standard deviation, whilst categorical variables were expressed as percentages. A *P* value <.05 was considered statistically significant. Analyses were conducted using Empower RCH (http://www.empowerstats.com). This cross-sectional study used receiver operating characteristic (ROC) curves, smooth curve fitting, multivariate regression analysis, and subgroup analysis to examine the association between depression, BMI, WC, WHtR, and WWI. In multivariate regression, Model 1: no covariates adjusted. Model 2: adjusted for age, gender, and race. Model 3: adjusted for age, gender, race, smoking history, drinking history, education level, marital status, PIR, history of diabetes, history of hypertension, HDL, total cholesterol, and fasting blood glucose. Key variables, including gender, age, and ethnicity, were analyzed in stratified subgroup analyses. The area under the ROC curve was calculated to evaluate the discriminatory ability of various body weight indicators (BMI, WC, WHtR, and WWI) for depressive status (PHQ-9 score ≥ 10 points).

## 3. Results

### 3.1. Baseline characteristics of the population

As shown in Table 1, 5042 participants were grouped into 4 categories based on PHQ-9 scores: (0–4), (5–9), (10–14), (15–27). Across depression severity groups, participants who were younger, female, less educated, single, had diabetes, hypertension, smoked, or abstained from alcohol exhibited higher depression levels (*P* < .05), indicating significant differences in baseline characteristics. Participants with lower poverty income ratio and HDL levels, and higher WC, WHtR, BMI, and WWI values exhibited greater depression severity (*P* < .05).

**Table 1:** Baseline characteristics of the population based on depression scores.

Variables	PHQ-9 (0–4)	PHQ-9 (5–9)	PHQ-9 (10–14)	PHQ-9 (15–27)	*P*
	N = 3416	N = 1002	N = 398	N = 226	
Age (yr)	55.45 ± 16.40	50.20 ± 17.71	47.86 ± 17.27	49.56 ± 17.65	<.001
Gender (%)					<.001
Male	1672 (48.95%)	394 (39.32%)	145 (36.43%)	87 (38.50%)	
Female	1744 (51.05%)	608 (60.68%)	253 (63.57%)	139 (61.50%)	
Race (%)					.056
Mexican American	212 (6.21%)	64 (6.39%)	27 (6.78%)	15 (6.64%)	
Other Hispanic	299 (8.75%)	125 (12.48%)	35 (8.79%)	28 (12.39%)	
NonHispanic White	2168 (63.47%)	590 (58.88%)	238 (59.80%)	127 (56.19%)	
NonHispanic Black	367 (10.74%)	117 (11.68%)	48 (12.06%)	28 (12.39%)	
Other race	370 (10.83%)	106 (10.58%)	50 (12.56%)	28 (12.39%)	
Education level (%)					<.001
<9th grade	104 (3.04%)	34 (3.39%)	17 (4.27%)	18 (7.96%)	
9–11th grade	220 (6.44%)	78 (7.78%)	25 (6.28%)	31 (13.72%)	
Highschool graduate	669 (19.58%)	205 (20.46%)	101 (25.38%)	51 (22.57%)	
Some college or AA degree	994 (29.10%)	342 (34.13%)	145 (36.43%)	88 (38.94%)	
College graduate or above	1429 (41.83%)	343 (34.23%)	110 (27.64%)	38 (16.81%)	
Marital status (%)					<.001
Married/living with partner	2055 (60.16%)	466 (46.51%)	171 (42.96%)	65 (28.76%)	
Widowed/divorced/separated	769 (22.51%)	280 (27.94%)	110 (27.64%)	73 (32.30%)	
Never married	592 (17.33%)	256 (25.55%)	117 (29.40%)	88 (38.94%)	
Diabetes (%)					<.001
Yes	412 (12.06%)	131 (13.07%)	75 (18.84%)	51 (22.57%)	
No	3004 (87.94%)	871 (86.93%)	323 (81.16%)	175 (77.43%)	
High blood pressure (%)					<.001
Yes	1230 (36.01%)	387 (38.62%)	131 (32.91%)	108 (47.79%)	
No	2186 (63.99%)	615 (61.38%)	267 (67.09%)	118 (52.21%)	
Alcohol use (%)					.026
Yes	2601 (76.14%)	778 (77.64%)	294 (73.87%)	155 (68.58%)	
No	815 (23.86%)	224 (22.36%)	104 (26.13%)	71 (31.42%)	
Smoking (%)					<.001
Yes	1290 (37.76%)	468 (46.71%)	191 (47.99%)	117 (51.77%)	
No	2126 (62.24%)	534 (53.29%)	207 (52.01%)	109 (48.23%)	
PIR	3.24 ± 1.52	2.80 ± 1.51	2.44 ± 1.50	2.07 ± 1.42	<.001
Fasting glucose (mg/dL)	108.95 ± 22.80	107.75 ± 18.07	111.29 ± 32.24	111.74 ± 33.48	.203
TC (mg/dL)	189.34 ± 39.99	188.07 ± 40.74	189.94 ± 44.21	190.56 ± 41.11	.494
HDL (mg/dL)	55.09 ± 14.44	54.45 ± 13.86	52.93 ± 12.62	52.84 ± 14.05	.012
WC (cm)	100.30 ± 15.87	102.54 ± 18.89	102.88 ± 18.13	104.66 ± 18.87	<.001
WHtR	0.60 ± 0.09	0.62 ± 0.11	0.62 ± 0.11	0.63 ± 0.11	<.001
BMI	29.18 ± 6.50	30.65 ± 7.87	30.92 ± 8.26	31.16 ± 8.33	<0.001
WWI	11.10 ± 0.82	11.17 ± 0.87	11.18 ± 0.88	11.38 ± 0.89	<0.001

BMI = body mass index, PHQ-9 = Patient Health Questionnaire 9, WC = waist circumference, WHtR = waist-to-height ratio, WWI = weight-adjusted-waist index.

### 3.2. Relationship between body mass index and depression scores

According to Table 2, in the multiple linear regression analysis, Model 1: no covariates adjusted. Model 2: adjusted for age, gender, and race. Model 3: adjusted for age, gender, race, smoking history, drinking history, education level, marital status, poverty income ratio, history of diabetes, history of hypertension, HDL, total cholesterol, and fasting blood glucose. WWI, WC, WHtR, and BMI were positively correlated with depression scores in Models 1, 2, and 3. In Model 3, every unit increase in WHtR, after controlling for all variables, was linked to a 413% rise in depression scores (β = 4.13, 95% confidence interval: 2.75–5.50, *P* < .001).

**Table 2:** Relationship between body mass index and depression scores.

Exposure	Model 1	Model 2	Model 3
	Nonadjusted	Adjust I	Adjust II
	β (95% CI) *P* value	β (95% CI) *P* value	β (95% CI) *P* value
WWI	0.40 (0.25, 0.55) <.0001	0.97 (0.80, 1.14) <.0001	0.57 (0.39, 0.75) <.0001
WC	0.02 (0.01, 0.03) <.0001	0.04 (0.03, 0.04) <.0001	0.02 (0.02, 0.03) <.0001
WHtR	4.89 (3.62, 6.15) <.0001	6.50 (5.22, 7.77) <.0001	4.13 (2.75, 5.50) <.0001
BMI	0.07 (0.05, 0.09) <.0001	0.07 (0.05, 0.09) <.0001	0.05 (0.03, 0.06) <.0001

Model 1: Nonadjusted (unadjusted for covariates), Model 2: Adjust I (adjusted for gender, age, and ethnicity), Model 3: Adjust II (adjusted for gender, age, ethnicity, education level, marital status, household income and poverty ratio, fasting blood glucose, HDL cholesterol, total cholesterol, alcohol consumption, smoking, diabetes, and hypertension).

BMI = body mass index, CI = confidence interval, HDL = high-density lipoprotein, WC = waist circumference, WHtR = waist-to-height ratio, WWI = weight-adjusted-waist index.

### 3.3. Subgroup analysis of obesity-related indicators and depression scores

Subgroup analysis revealed statistically significant gender and age variations in the relationship between WC, WHtR, and depression scores. The association between WWI, BMI, and depression ratings did not show statistically significant variations by gender or age, as shown in Table 3.

**Table 3:** Subgroup analysis of weight-related indices and depression scores.

	WHtR	*P*	WC	*P*	WWI	*P*	BMI	*P*
Gender		.0350		.0146		.0691		.0746
Male	2.34 (0.25, 4.42) 0.0283		0.01 (0.00, 0.02) 0.0326		0.35 (0.08, 0.63) 0.0124		0.02 (−0.01, 0.05) 0.1185	
Female	5.09 (3.39, 6.78) <0.0001		0.03 (0.02, 0.04) <0.0001		0.67 (0.45, 0.89) <0.0001		0.06 (0.03, 0.08) <0.0001	
Age		.0317		.0197		.1212		.0542
20–39	5.70 (3.16, 8.23) <0.0001		0.04 (0.02, 0.05) <0.0001		0.70 (0.37, 1.04) <0.0001		0.07 (0.03, 0.10) 0.0002	
40–59	1.32 (−1.15, 3.78) 0.2958		0.01 (−0.01, 0.02) 0.3623		0.23 (−0.10, 0.55) 0.1687		0.01 (−0.02, 0.05) 0.4415	
≧60	4.83 (2.61, 7.04) <0.0001		0.03 (0.01, 0.04) <0.0001		0.53 (0.25, 0.80) 0.0002		0.06 (0.03, 0.09) 0.0002	

Race, smoking history, drinking history, education level, marital status, PIR, history of diabetes, history of hypertension, HDL, TC, and fasting blood glucose were adjusted.

BMI = body mass index, HDL = high-density lipoprotein, PIR = poverty income ratio, TC = total cholesterol, WC = waist circumference, WHtR = waist-to-height ratio, WWI = weight-adjusted-waist index.

### 3.4. Smooth curve fitting and threshold effect analysis of obesity-related indicators and depression scores

**Figure 2.**
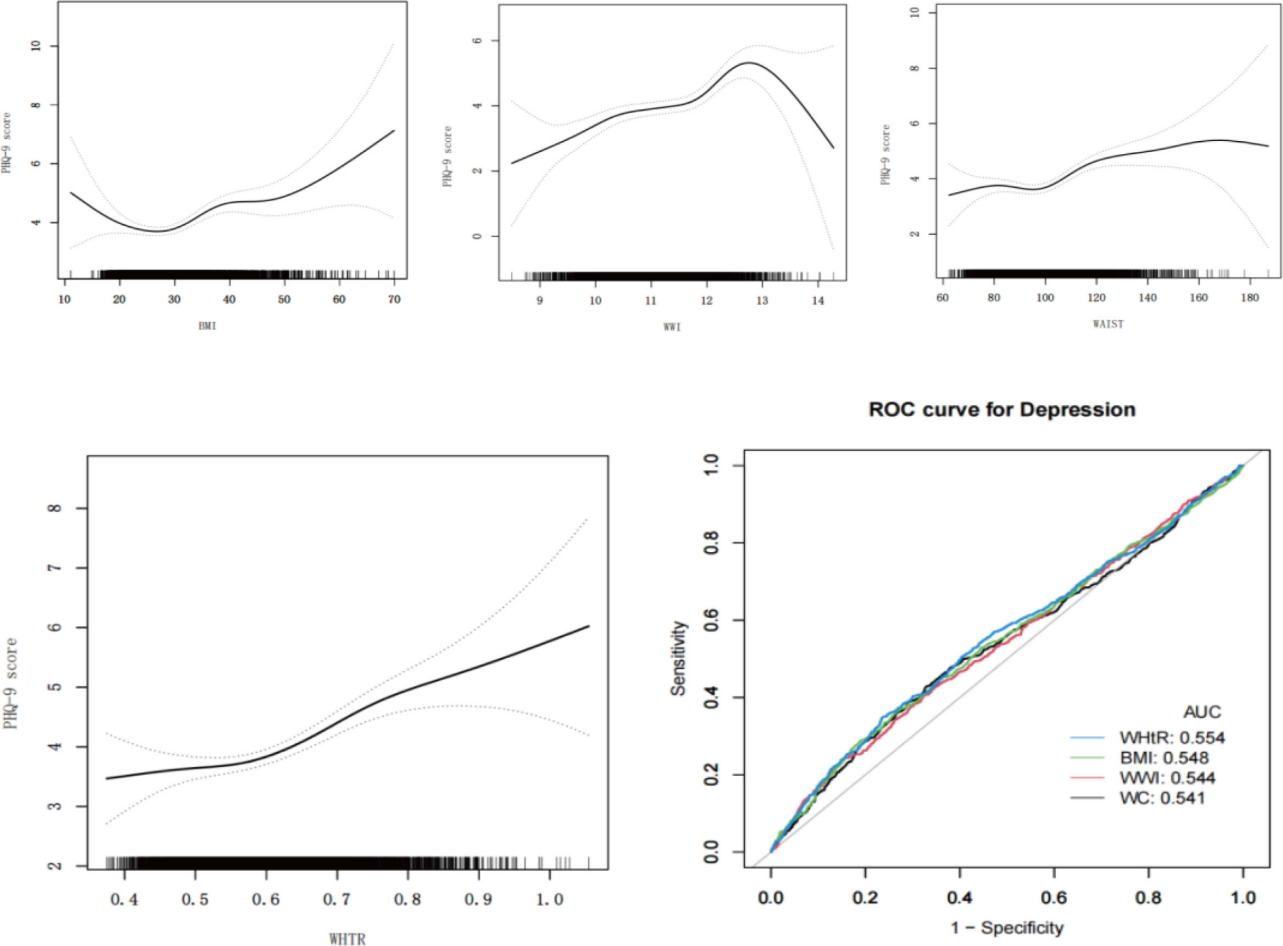
Smooth curve fitting and area under the curve for WWI, WHtR, WC, BMI, and depression. AUC = area under the curve, BMI = body mass index, PHQ-9 = Patient Health Questionnaire 9, ROC = receiver operating characteristic, WC = waist circumference, WHtR = waist-to-hip ratio, WWI = weight-adjusted waist index.

Figure 2 and Table 4 show smooth curve fitting revealing nonlinear relationships between WC, WHtR, BMI, and depression scores. WC, WHtR, and BMI exhibited statistically significant correlations beyond the breakpoint, with WHtR demonstrating a strong association (odd ratio = 5.92, 95% confidence interval: 3.97–7.87). Participant ROC analysis results indicated ROC values of 0.554, 0.548, 0.544, and 0.541 for WHtR, BMI, WWI, and WC, respectively.

**Table 4:** Threshold effect analysis of weight-related indices and depression scores.

	WHtR	WC	WWI	BMI
	β (95% CI) *P*	β (95% CI) *P*	β (95% CI) *P*	β (95% CI) *P*
Model 1	4.13 (2.75, 5.50) <.0001	0.02 (0.02, 0.03) <.0001	0.57 (0.39, 0.75) <.0001	0.05 (0.03, 0.06) <.0001
Model 2				
Breakpoint (*K*)	0.58	97.6	11.82	27.6
OR1	−0.08 (−3.62, 3.45) .9633	−0.00 (−0.02, 0.02) .7174	0.48 (0.26, 0.71) <.0001	−0.05 (−0.10, 0.01) .1192
OR2	5.92 (3.97, 7.87) <.0001	0.04 (0.02, 0.05) <.0001	0.88 (0.35, 1.41) .0012	0.07 (0.05, 0.10) <.0001
*P* for log likelihood ratio	.011	.004	.216	.001

Age, gender, race, smoking history, drinking history, education level, marital status, PIR, history of diabetes, history of hypertension, HDL, TC, and fasting blood glucose were adjusted.

BMI = body mass index, CI = confidence interval, HDL = high-density lipoprotein, OR = odds ratio, PIR = poverty income ratio, TC = total cholesterol, WC = waist circumference, WHtR = waist-to-height ratio, WWI = weight-adjusted-waist index.

## 4. Discussion

The results of this 5042-person cross-sectional survey revealed significant positive correlations between BMI, WC, WHtR, and depression scores. Nonlinear relationships were observed between WC, WHtR, BMI, and PHQ-9 scores, with stronger associations when WHtR exceeded 0.58. Gender and age differences showed no statistically significant effects on the connection between WWI and BMI with depression scores. The predictive power of WWI, WC, WHtR, and BMI for adult depression was comparable.

Obesity and depression influence each other through a bidirectional relationship. Increased body weight triggers inflammatory factors, disrupting the balance of the hypothalamic-pituitary-adrenal axis and the leptin-melanocortin system. Emotionally, this leads to self-denial, social withdrawal, and self-isolation, intensifying feelings of loneliness and depression. Conversely, depression disrupts dietary patterns, reduces physical activity and energy expenditure, and is accompanied by insomnia or hypersomnia. Sleep disturbances further disrupt the balance of leptin and ghrelin, exacerbating obesity.^[13,14]^ Jan Debski et al suggest that weight loss achieved through increased physical activity may help reduce depressive symptoms.^[9]^ Depression is currently understood as a result of the combined effects of genetic susceptibility, neurotransmitter and endocrine hormone dysregulation, inflammatory states, as well as cognitive biases, personality traits, and psychological trauma. Clinical treatment targets remain unclear, and therapeutic outcomes are suboptimal.^[15]^ Shiyi Tao et al found that Drew D. Decker et al demonstrated that a low-carbohydrate ketogenic diet pattern, which improves metabolic health, can adjunctively treat depressive symptoms, indirectly reflecting the role of metabolic indicators in depression.^[14,16]^ Shipan Zhang et al found that the triglyceride-to-glucose index and BMI index are risk factors for depression. Considering that insulin resistance itself stems from imbalances in diet, exercise, and obesity, this also demonstrates that improving weight-related indicators can lessen the incidence of depression.^[17]^

Obesity is the result of dietary imbalance, reduced physical activity, and hormonal disorders. Central obesity accounts for the majority of obese individuals and is a stronger predictor of diabetes and cardiovascular event risk.^[12,18]^ BMI is primarily used to assess obesity, while WC, WHtR, and WWI serve as indicators of central obesity. Combined, these 3 metrics comprehensively capture both the “total accumulation” and “distribution abnormalities” of fat deposits. Some researchers also suggest that WWI reflects the relationship between muscle and fat. Yali Guo et al discovered that WWI is connected to an increased risk of developing depression.^[6,8]^ Nam Hoon Kim et al discovered that elevated WWI in older people correlates with greater fat mass and reduced muscle mass, reflecting the relationship between muscle and fat in the human body.^[19]^

According to subgroup analysis results, the effects of WWI and BMI on depression demonstrated stability and were not easily moderated by gender and age factors. It identified critical thresholds at WC 97.6 cm, WHtR 0.58, and BMI 27.6 kg/m^2^, where the risk of depression transitions from “slow accumulation” to “accelerated escalation.” Precise interventions at these qualitative change points may potentially halt the progression toward adult depression. Both obesity and depression are linked to hormonal factors. Hormonal fluctuations during the menstrual cycle, the abrupt hormonal decline during menopause, and a higher prevalence of thyroid disorders may collectively elevate women’s depression risk. Different age groups face distinct pressures: work and marital stress in adulthood, physiological aging in later life, diminished emotional regulation, chronic illnesses, and solitary living may all contribute to depression.^[20]^

The strength of this study lies in its systematic comparison of 4 obesity indicators within a nationally representative sample of U.S. adults, providing evidence for selecting more valuable obesity screening tools in clinical and public health practice. This study also has its drawbacks. Due to the cross-sectional design, causality cannot be established; therefore, we cannot determine whether higher WHtR, BMI, WWI, or WC levels lead to depressive progression. The PHQ-9 questionnaire is highly subjective and may introduce bias, potentially skewing depression assessments. Further investigation of potential biological mechanisms requires larger-scale studies, such as animal experiments.

## 5. Conclusion

The findings of this study indicate that body weight indicators – WWI, WC, WHtR, and BMI – are associated with depression risk. Key thresholds were identified, including WC 97.6 cm, WHtR 0.58, and BMI 27.6 kg/m^2^, and the impact of BMI on depression remained stable across different gender and age subgroups. Lifestyle interventions targeting these indicators may effectively prevent and mitigate the progression of depression.

## Acknowledgments

We express our appreciation to the participants and staff of the NHANES project.

## Author contributions

**Conceptualization:** Wenting Deng, Jian Mei.

**Data curation:** Wenting Deng.

**Formal analysis:** Wenting Deng.

**Investigation:** Wenting Deng.

**Methodology:** Wenting Deng, Jian Mei.

**Software:** Wenting Deng.

**Supervision:** Wenting Deng.

**Validation:** Wenting Deng.

**Visualization:** Wenting Deng.

**Writing – original draft:** Wenting Deng.

**Writing – review & editing:** Wenting Deng.
